# Fronto-temporal white matter connectivity predicts reversal learning errors

**DOI:** 10.3389/fnhum.2015.00343

**Published:** 2015-06-18

**Authors:** Kylie H. Alm, Tyler Rolheiser, Feroze B. Mohamed, Ingrid R. Olson

**Affiliations:** ^1^Department of Psychology, Temple University, Philadelphia, PAUSA; ^2^Department of Radiology, Temple University School of Medicine, Philadelphia, PAUSA

**Keywords:** orbitofrontal cortex, hippocampus, uncinate fasciculus, diffusion imaging, reversal learning, decision making

## Abstract

Each day, we make hundreds of decisions. In some instances, these decisions are guided by our innate needs; in other instances they are guided by memory. Probabilistic reversal learning tasks exemplify the close relationship between decision making and memory, as subjects are exposed to repeated pairings of a stimulus choice with a reward or punishment outcome. After stimulus–outcome associations have been learned, the associated reward contingencies are reversed, and participants are not immediately aware of this reversal. Individual differences in the tendency to choose the previously rewarded stimulus reveal differences in the tendency to make poorly considered, inflexible choices. Lesion studies have strongly linked reversal learning performance to the functioning of the orbitofrontal cortex, the hippocampus, and in some instances, the amygdala. Here, we asked whether individual differences in the microstructure of the uncinate fasciculus, a white matter tract that connects anterior and medial temporal lobe regions to the orbitofrontal cortex, predict reversal learning performance. Diffusion tensor imaging and behavioral paradigms were used to examine this relationship in 33 healthy young adults. The results of tractography revealed a significant negative relationship between reversal learning performance and uncinate axial diffusivity, but no such relationship was demonstrated in a control tract, the inferior longitudinal fasciculus. Our findings suggest that the uncinate might serve to integrate associations stored in the anterior and medial temporal lobes with expectations about expected value based on feedback history, computed in the orbitofrontal cortex.

## Introduction

A classic task used to assess stimulus–response learning and cognitive flexibility is reversal learning. In this task, non-human animals or human participants learn to associate various stimuli with either rewards or penalties. Once these associations are adequately learned, the corresponding reward contingencies are reversed, and participants must learn to respond to the newly rewarded stimulus, while suppressing responses to the previously rewarded stimulus. Participants are not immediately aware of this reversal, so the previously rewarded stimulus remains pre-potent. The ease (or difficulty) with which participants learn to adapt their choices based on the reversal phase indexes cognitive flexibility. Reversal learning can be thought of as a type of *model-based learning* (Lucantonio et al., 2012) in which the incoming reward and punishment information is used to update model parameters and make decisions.

Individual differences in the propensity toward perseveration after reversal reveal important differences in the tendency to make inflexible, and perhaps impulsive choices (Clark et al., 2004). Indeed, reversal learning deficits have been observed across a range of psychiatric disorders that share a core propensity for inflexible and impulsive decision making: certain types of substance abuse (Jentsch et al., 2002; Obernier et al., 2002; Ersche et al., 2008; Fortier et al., 2008; Schoenbaum and Shaham, 2008; Crews and Boettiger, 2009; de Ruiter et al., 2009; Stalnaker et al., 2009; Izquierdo and Jentsch, 2012; Ortega et al., 2013), attention deficit hyperactivity disorder (Kempton et al., 1999; Itami and Uno, 2002), bipolar disorder (McKirdy et al., 2009; Roiser et al., 2009; Dickstein et al., 2010), conduct disorder (Budhani and Blair, 2005), and psychopathy (Mitchell et al., 2002; Budhani et al., 2006).

Investigations into the neural basis of reversal learning have strongly implicated several highly interconnected limbic and paralimbic brain regions: the orbitofrontal cortex (OFC), the hippocampus, and the amygdala. A rich literature of neuroimaging, neuropsychological, and lesion studies suggests that specific reward contingencies are encoded in the OFC, which is also responsible for maintaining and updating the reward history associated with particular stimuli (Clark et al., 2004; Frank and Claus, 2006; Fellows, 2011). Damage to the OFC leads to severe impairments on exceedingly simple reversal tasks across rodents, monkeys, and humans (Fellows and Farah, 2003; Frank and Claus, 2006; Fellows, 2011; Izquierdo and Jentsch, 2012). Similar findings have emerged in patients with hippocampal lesions. Evidence suggests that after hippocampal damage, patients exhibit intact initial acquisition of stimulus–outcome associations; however, performance is impaired when they are required to reverse the associations (Shohamy et al., 2009). This has been demonstrated across human patients, rodents, rabbits, and monkeys (Kimble and Kimble, 1965; Berger and Orr, 1983; Whishaw and Tomie, 1997; Murray et al., 1998; Myers et al., 2000, 2006; Carrillo et al., 2001; Shohamy et al., 2009). Similar reversal deficits have also been documented after damage to the amygdala (Schwartzbaum and Poulos, 1965; Aggleton and Passingham, 1981; Izquierdo and Murray, 2004). Therefore, researchers have posited that the neural regions within this OFC-hippocampus-amygdala circuit contribute to successful reversal learning in similar manners.

However, this view has recently been challenged. It has been proposed that damage to the white matter connecting the OFC to the temporal lobes is the source of reversal learning deficits, rather than damage to OFC gray matter. Specifically, Rudebeck et al. (2013) trained adult rhesus monkeys to learn which of two objects was associated with a food reward. After reaching a criterion, the objects’ associated reward contingencies were reversed and training with the reversed contingencies continued until the criterion was reached again. Results revealed that monkeys with excitotoxic lesions to the OFC, which are restricted to cortical gray matter, exhibited normal reversal learning performance, as measured by errors to criterion. By contrast, monkeys with aspiration lesions to the OFC, which encompass both white and gray matter, made significantly more errors before reaching the criterion, revealing consistent impairments in reversal learning relative to both controls and animals that received excitotoxic lesions. This finding was replicated in a follow-up experiment using smaller aspiration lesions restricted to the posterior portion of the OFC.

A similar paradox has also been observed after amygdala damage. Several more recent studies (e.g., Izquierdo and Murray, 2007; Stalnaker et al., 2007; Rudebeck and Murray, 2008; Izquierdo et al., 2013) have demonstrated intact or even improved reversal learning performance after amygdala damage, which stands in contrast to prior evidence linking amygdala damage to reversal learning deficits (Schwartzbaum and Poulos, 1965; Aggleton and Passingham, 1981; Izquierdo and Murray, 2004). Even an early account of hippocampal reversal deficits pointed out that the lesions that were created usually included white matter of the surrounding temporal cortex, making it difficult to disentangle the contributions of gray matter versus white matter (Berger and Orr, 1983). It has thus been proposed (but not demonstrated) that damage to the white matter connecting the OFC to the medial temporal lobes (MTLs) may be the source of reversal learning deficits, rather than damage to the OFC or MTL gray matter *per se* (Baxter and Croxson, 2013; Rudebeck et al., 2013).

Here, we tested a related idea by examining the relationship between reversal learning performance and microstructural properties of a white matter tract called the *uncinate fasciculus* (UF; Catani and Thiebaut de Schotten, 2008). This tract forms a monosynaptic pathway between regions in the anterior and medial temporal lobes (the temporal pole, uncus, parahippocampal gyrus – including the entorhinal and perirhinal cortices, and amygdala) and regions in the frontal lobe (the lateral OFC and frontal pole; Catani et al., 2013; Von Der Heide et al., 2013). By virtue of its location and connectivity (i.e., connections to MTL regions and the amygdala), the UF is considered a limbic pathway. However, the function(s) and information transmission properties of the UF are largely unknown. Several diffusion imaging studies have posited a relationship between the UF and episodic memory functions (Diehl et al., 2008; McDonald et al., 2008; Niogi et al., 2008; Metzler-Baddeley et al., 2011), decision making (e.g., Camara et al., 2010), and several psychiatric disorders, including anxiety (Phan et al., 2009; Baur et al., 2013, 2012). Our review of the non-human primate lesion literature and human diffusion imaging literature led us to hypothesize that the UF plays a role in decision making, as well as long-term memory processing, by integrating associations stored in the MTLs with feedback history computed in the OFC (Von Der Heide et al., 2013). If true, individual differences in UF microstructure should predict the ability to flexibly adapt to changes in reward contingencies, a key part of reversal learning. As a control task, we included a go/no-go task, which requires inhibition of pre-potent responses but does not require the retrieval of stored memory representations to perform the task well. As a control white matter tract, we examined the inferior longitudinal fasciculus (ILF). The ILF is similar to the UF in that it enters the medial and anterior temporal lobes. However, its function is more closely aligned with high level vision, and it lacks any relationship to the frontal lobe (Catani et al., 2003; Pyles et al., 2013).

We used diffusion tensor imaging (DTI), along with deterministic tractography, in order to examine the microstructural properties of the UF and ILF. DTI utilizes diffusion-weighted MR imaging (DW-MRI) to measure the degree of diffusion of the water molecules within human brain tissue. In myelinated axons, which make up white matter tracts, the direction of diffusion is restricted due to the presence of myelin sheaths, neurofilaments, microtubules, and cell membranes (Beaulieu, 2002). DW-MRI captures the degree of restriction, called anisotropy, and provides measures of the microstructural properties of white matter, such as the orientation and magnitude of diffusion within each voxel of the brain (Alexander et al., 2007, 2011; Jones, 2008; Tournier et al., 2011). Tractography allows for the visualization of white matter tracts and can be combined with DTI to calculate microstructural indices specific to particular white matter tracts.

## Materials and Methods

### Participants

Thirty-five healthy individuals (14 male, 21 female) between the ages of 18 and 28 (*M* = 21.51, SD = 2.55) volunteered for this experiment. Two substantial outliers were found across white matter indices and thus, two participants were excluded from further analyses, leaving a study sample of 33 participants. All participants were right-handed, native English speakers with normal to corrected-to-normal vision. All participants had no history of psychological or neurological disorders as ascertained by self-report and no MRI contraindications. Informed consent was obtained according to the guidelines of the Institutional Review Board of Temple University, and participants received monetary compensation for participation in the experiment.

### Study Protocol

Study participation occurred in two separate testing sessions. During the behavioral session, participants completed computerized tasks in the laboratory. Participants were tested individually in a well-lit room. Computerized tasks were programmed in E-Prime (Version 2.0 Professional) and presented on Dell computers. During a separate scanning session, DTI data, as well as high-resolution anatomical scans, were acquired at Temple University Hospital.

#### Computerized Iowa Gambling Task with Reversal

Reversal learning was assessed using a modified version of the Iowa Gambling Task (IGT; Bechara et al., 1994). Participants were instructed to choose freely from four decks of cards, presented on a computer screen, in an attempt to accrue as many points as possible. They were informed that each deck was associated with both rewards (positive points) and penalties (negative points). The point values gained or lost on each trial, along with the running total, were displayed after each choice. Reward probabilities followed the standard IGT design (Bechara et al., 1994). Briefly, two decks yielded higher immediate rewards but were also associated with frequent small to moderate losses, or less frequent but substantial losses. These decks returned a net loss and were considered the disadvantageous decks. The remaining two decks, the advantageous decks, yielded lower immediate rewards but only moderate or infrequent losses and returned a net gain. These contingencies were not disclosed to participants; however, participants were told to keep in mind that some decks may be “better” than other decks and more points would be earned if they could identify the “better” decks. Timing was self-paced.

After 200 trials of initial acquisition of the associated reward contingencies, the reward/penalty contingencies associated with each deck were reversed such that the classic advantageous decks became decks that yielded a net loss (newly disadvantageous), while the classic disadvantageous decks became the decks that yielded a net gain (newly advantageous). This constituted the reversal phase and lasted 200 trials. The entire task lasted approximately 15 minutes. Overall performance was examined by calculating net IGT score, a commonly used measure which subtracts the sum of disadvantageous deck choices from the sum of advantageous deck choices for each participant. Additionally, reversal learning was measured by calculating the total number of reversal errors committed by each participant. A reversal error consisted of any trial where participants chose from either of the previously advantageous decks after the contingency reversal occurred.

#### Go/No-Go Task

A subset of the study sample (*n* = 24) completed a go/no-go task in order to assess response inhibition abilities. Participants focused on a central fixation cross while random letters appeared one at a time in the center of the screen. Participants were instructed to press the space bar as quickly as possible whenever they saw a letter appear on the screen (go trials). However, any time the letter “X” appeared on the screen, participants were told to withhold their response (no-go trials). The task consisted of 128 total trials; 103 go trials and 25 pseudorandomly placed no-go trials. Performance was assessed by calculating *d*′, a measure of discrimination sensitivity, by subtracting normalized false alarm rate from normalized hit rate.

### Image Acquisition

MRI scanning was conducted at Temple University Hospital on a 3.0 T Siemens Verio scanner (Erlangen, Germany) using a conventional 12-channel phased-array head coil. DTI data were collected using a diffusion-weighted echo-planar imaging (EPI) sequence covering the whole brain. Salient imaging parameters were as follows: 55 axial slices, 2.5 mm slice thickness, Repetition Time (TR) = 9,900 ms, Echo Time (TE) = 95 ms, Field of View (FOV) = 240 mm × 240 mm, *b*-values of 0 and 1000 s/mm^2^, 64 non-collinear directions. These parameters yielded a DTI scan lasting approximately 11 minutes.

In addition to diffusion-weighted images, high-resolution anatomical images (T1-weighted 3D MPRAGE) were also collected for each participant with the following parameters: 160 axial slices, 1 mm slice thickness, TR = 1,900 ms, TE = 2.93 ms, inversion time = 900 ms, flip angle = 9°, FOV = 256 mm × 256 mm. These anatomical images were co-registered to the diffusion images and used to draw regions of interest (ROIs).

### DTI Pre-Processing

The diffusion-weighted data were pre-processed using FSL (Smith et al., 2004) to correct for eddy currents and subject motion using an affine registration model. The b-vector matrix was adjusted based on rigid body registration, ensuring a valid computation of the tensor variables. Non-brain tissue was removed using FSL’s (Smith et al., 2004) automated brain extraction tool (BET), and a standard diffusion tensor fitting model was then applied to the data. The diffusion tensor fitting provided estimates of fractional anisotropy (FA) and mean diffusivity (MD), as well as three eigenvectors and eigenvalues. These estimates were computed on individual voxels using a three-dimensional Gaussian distribution model that yielded a single mean ellipsoid for each voxel.

Whole brain deterministic tractography was performed in subject native space using the Diffusion Toolkit and TrackVis software packages (Wang et al., 2007). This software uses a fiber assignment continuous tracking (FACT) algorithm (Mori et al., 1999) to determine the branching and curving of the fiber tracts. For a given voxel, this algorithm estimates the orientation of the principal eigenvector in that voxel and then uses nearest-neighbor interpolation to step along that direction. Step length was fixed at 0.1 mm, and an angle threshold of 35° was used to determine the termination point of the fiber tracts. A spline filter was used to smooth the tractography data. A multiple ROI-based axonal tracking approach (Mori et al., 2002; Wakana et al., 2007; Thomas et al., 2010) was then used to delineate bilateral UFs by drawing seed regions in the anterior temporal lobe and OFC. ROIs were drawn in subject native space using the high-resolution anatomical T1 images and the methods outlined by Thomas et al. (2010). A Boolean AND term was used to select only the fibers that passed through both of these seed ROIs. A control tract, the ILF, was also traced bilaterally using the same methodology and the ROIs outlined by Thomas et al. (2010). MD and axial diffusivity (AD) indices were subsequently extracted from the tracts of interest, averaging along the length of each tract.

In this study, we specifically focused on the indices of MD and AD. MD is a measure of the overall degree of diffusion, regardless of direction, while AD is a measure of the degree of diffusion along the axis parallel to the axon fibers (Beaulieu, 2011). We chose these two white matter indices because they are thought to reflect more specific information regarding the underlying microstructural properties of white matter (Alexander et al., 2011, 2007). Further, although FA is the most commonly reported microstructural measure, certain studies suggest that FA may be a suboptimal measure for capturing the complexities of the underlying cellular architecture, as it correlates poorly with actual values of individual fiber anisotropy (Leow et al., 2009; Acosta-Cabronero et al., 2010).

### Statistical Analyses

Statistical analyses were performed using SPSS (Version 21.0). Regression analyses were used to examine the relationship between microstructure of the UF and performance on the IGT. Gender differences were found within some of the white matter indices, so it was controlled for in the regressions. To control for multiple comparisons, family-wise error rate was adjusted using a Bonferroni correction to account for simultaneous predictors in each regression model (critical *p* = 0.05/3; Mundfrom et al., 2006). All regression *p*-values reported are Bonferroni-corrected.

## Results

### Behavioral Data

**FIGURE 1:**
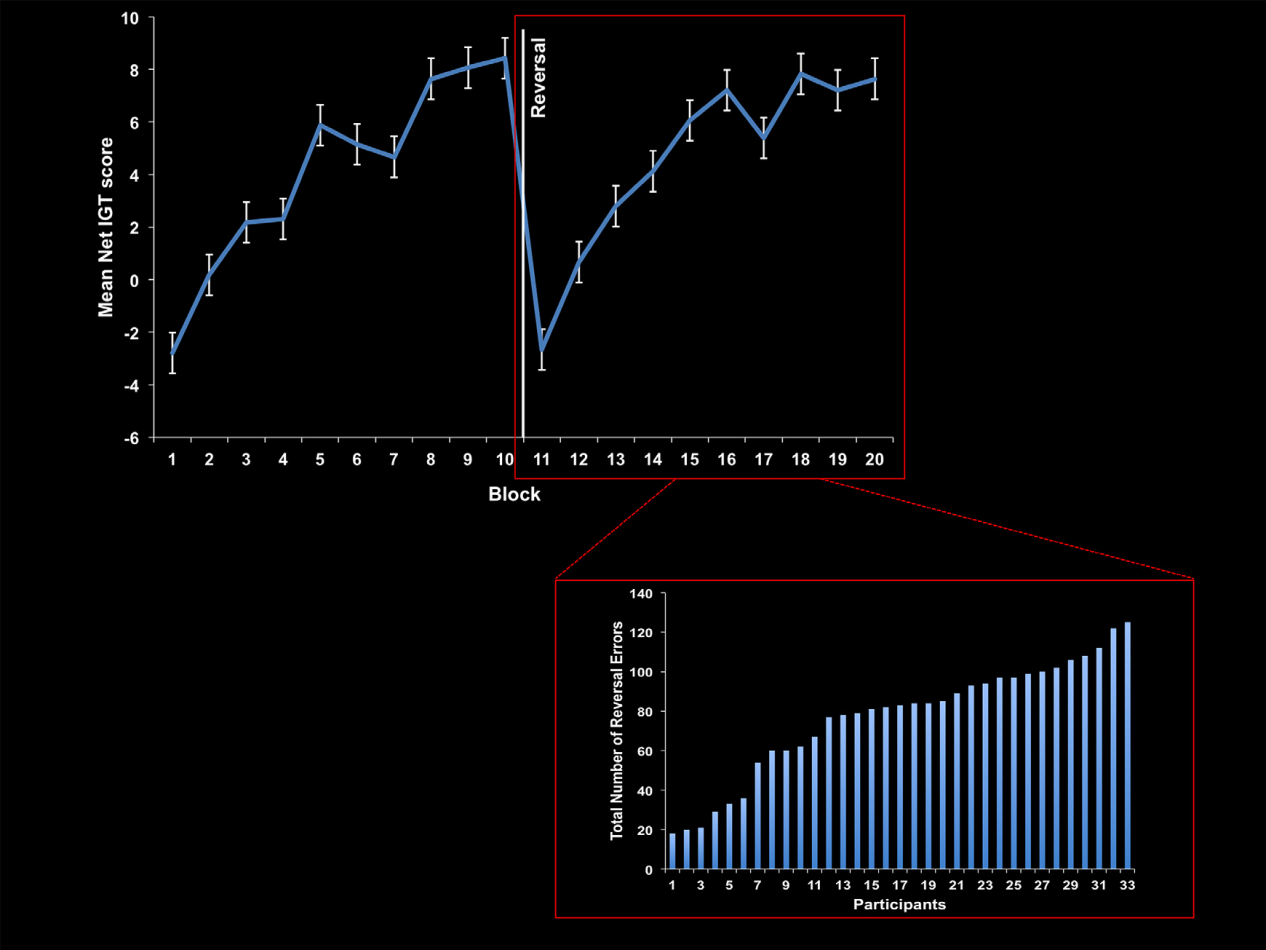
**Behavioral performance on the Iowa Gambling Task (IGT).** Group performance is plotted across test blocks **(top)**. IGT net score reflects the sum of disadvantageous deck choices subtracted from the sum of advantageous deck choices. Error bars represent standard error of the mean. Specific to the reversal phase, the total number of reversal errors committed is plotted for each participant **(bottom)** to display the individual variability among participants.

Behavioral results are presented in **Table 1** and **Figure 1**. Consistent with prior findings, participants’ performance on the IGT+reversals improved significantly from the first block to the last block on both the acquisition [*t*(32) = 4.55, *p* < 0.001] and reversal phases [*t*(32) = 4.07, *p* < 0.001] of the IGT.

**Table 1:** Mean performance on behavioral tasks.

	Mean (SD)
IGT acquisition phase net score	41.70 (75.75)
IGT reversal phase errors	76.88 (29.55)
Go/no-go d′	3.00 (0.67)

*IGT, Iowa Gambling Task*.

Thus, participants were able to learn the respective reward contingencies and later, update those reward contingencies after the reversal. However, there were individual differences in learning performance, especially during the reversal phase (see **Figure 1**). Some participants rapidly adapted to the altered contingencies, while others perseverated for many trials.

### DTI Data

The UF has a late maturational profile, extending throughout adolescence and young adulthood (Lebel et al., 2012). Therefore, we examined whether age effects were apparent in our relatively small sample. Regression analyses revealed that age was not significantly predicted by any microstructural indices of the UF or ILF (all *p’s* > 0.30). These analyses are presented in the Supplementary Table S1.

Recent studies have reported gender differences in DTI measures (Gong et al., 2011; Kanaan et al., 2012; but see Hänggi et al., 2014); therefore, we investigated potential differences in our sample. *T*-tests revealed that males exhibited decreased AD in the left UF, compared to females, *t*(31) = 2.43, *p* = 0.02. However, males exhibited increased AD [*t*(31) = 2.80, *p* = 0.009] and increased MD [*t*(31) = 2.11, *p* = 0.04] in the left ILF, relative to females. Results are presented in **Table 2**. Importantly, no gender differences were found with respect to behavioral performance measures. However, since we observed gender differences in white matter indices, this was included as a control variable in all subsequent analyses.

**Table 2:** Relationship between gender and microstructural properties of the UF **(A)** and ILF **(B)**.

(A)
	**Left UF**			**Right UF**		
	**Males**	**Females**	***p*-value**	**Males**	**Females**	***p*-value**

AD (×10^-3^ mm^2^/s)	1.144	1.182	0.02*	1.137	1.142	0.80
MD (×10^-3^ mm^2^/s)	0.740	0.774	0.11	0.736	0.742	0.68

**(B)**						
	**Left ILF**			**Right ILF**		
	**Males**	**Females**	***p*-value**	**Males**	**Females**	***p*-value**

AD (×10^-3^ mm^2^/s)	1.345	1.255	0.009**	1.249	1.264	0.62
MD (×10^-3^ mm^2^/s)	0.809	0.765	0.04*	0.758	0.777	0.29

*p < 0.05, **p < 0.01. UF, uncinate fasciculus; ILF, inferior longitudinal fasciculus; AD, axial diffusivity; MD, mean diffusivity.

### Is the Uncinate Fasciculus Involved in Reversal Learning as Tested by the IGT?

Regression models were constructed to predict total number of reversal errors committed during the reversal phase and net IGT score during the acquisition phase of the IGT. Predictors consisted of bilateral white matter indices (MD and AD) and gender, which was included as a control variable. Regression analyses revealed a relationship between individual differences in mean uncinate AD and performance during the reversal phase, but not performance during the initial acquisition phase of the IGT. This relationship was specific to the left UF. More precisely, after controlling for gender, individual differences in left UF AD significantly predicted the total number of reversal errors committed [β = −0.57, *t*(29) = 2.65, *p* = 0.039], but did not predict performance during the acquisition phase of the IGT [β = 0.24, *t*(29) = 1.02, *p* = 0.95]. The regression results are presented in **Table 3**. To directly compare the magnitudes of the left uncinate and ILF model predictors, a *z*-test was performed using the respective regression coefficients (Paternoster et al., 1998). The magnitude of the relationship between reversal errors and left uncinate AD was significantly greater than the magnitude of the relationship between reversal errors and left ILF AD (*z* = 2.07, *p* = 0.038).

**Table 3:** Summary of multiple linear regression models predicting performance on the acquisition and reversal phases of the IGT.

		Uncinate Fasciculus (UF)				Inferior Longitudinal Fasciculus (ILF)			
Dependent variable	Predictor variables	β	*t*-value	*F*	*R^2^*	β	*t*-value	*F*	*R^2^*
Acquisition net score				0.88	0.08			0.08	0.01
	Gender	0.16	0.82			0.07	0.31		
	Left AD	0.24	1.02			-0.02	0.09		
	Right AD	0.09	0.42			-0.06	0.33		
Acquisition net score				0.27	0.03			0.09	0.01
	Gender	0.11	0.57			0.09	0.42		
	Left MD	0.17	0.81			-0.08	0.37		
	Right MD	-0.04	0.18			-0.02	0.09		
Reversal errors				2.79*	0.22			0.62	0.06
	Gender	-0.40	2.20			-0.11	0.54			
	**Left AD**	-**0.57**	2.65*			-0.18	0.86		
	Right AD	0.25	1.28			-0.03	0.15		
Reversal errors				1.87	0.16			1.50	0.14
	Gender	-0.29	1.62			-0.10	0.52		
	Left MD	-0.42	2.10			-0.30	1.59		
	Right MD	0.21	1.09			-0.10	0.57		

*p < 0.05 (after correcting for multiple comparisons). Bold font is used to highlight the significant findings. IGT, Iowa Gambling Task; AD, axial diffusivity; MD, mean diffusivity; β, standardized regression coefficient.

**FIGURE 2:**
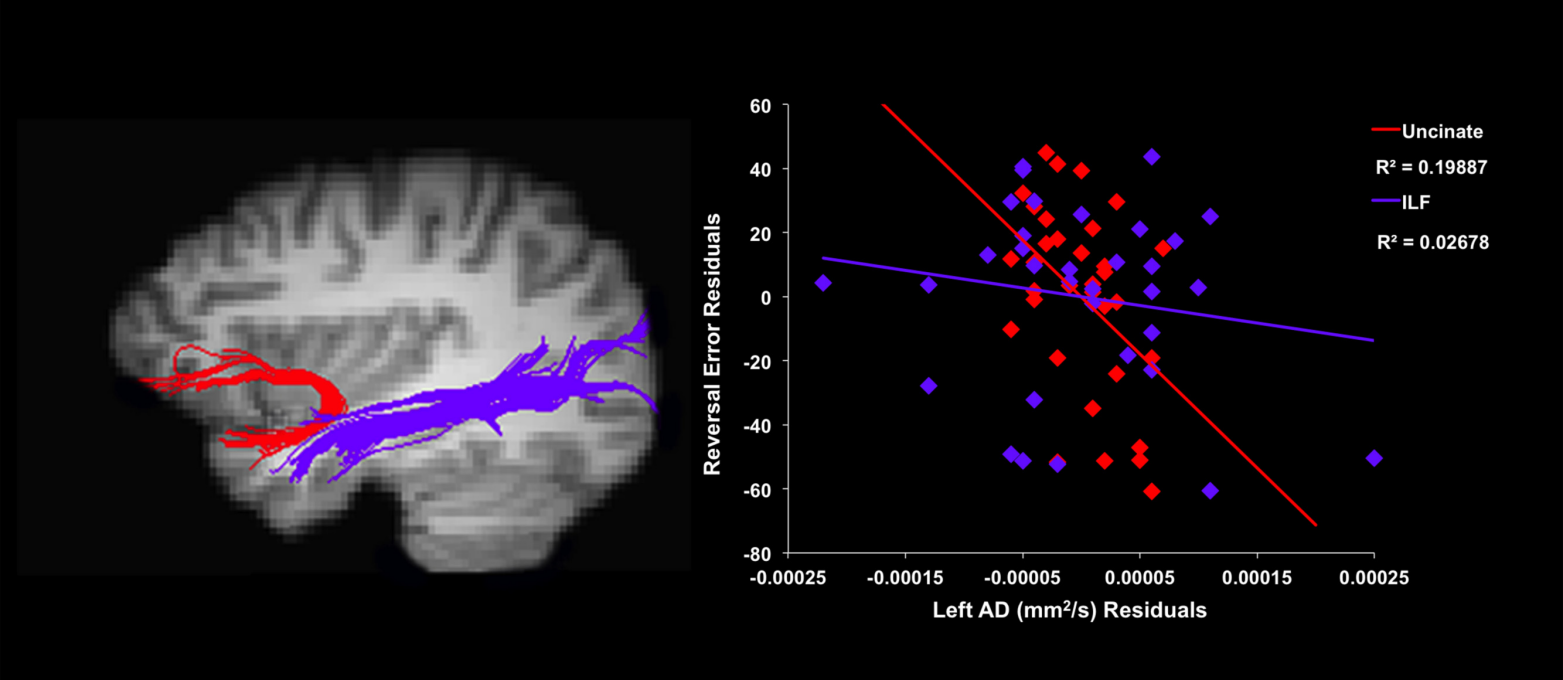
**Tractography delineating the uncinate fasciculus (UF; red) and inferior longitudinal fasciculus (ILF; purple) in a sample participant (left)**. Scatter plots of residuals from the linear regression analyses **(right)** illustrating the relationship between individual differences in reversal errors and mean left axial diffusivity (AD) of the respective white matter tracts.

**Figure 2** depicts the partial regression plot representing the unique contribution of left UF AD to the prediction of reversal errors. These findings suggest a negative relationship between left uncinate AD and reversal learning, such that lower values of AD were associated with a higher number of reversal errors. Right uncinate AD did not significantly predict performance on either phase of the IGT (*p’s* > 0.60). Similarly, neither relationship was significant for right uncinate MD (*p’s* > 0.14). Left uncinate MD did predict number of reversal errors; however, this effect did not survive correction for multiple comparisons (*p* = 0.05 uncorrected). Similarly, follow-up analyses were completed to examine the same relationships using radial diffusivity (RD). Uncorrected, there was a trending relationship between left uncinate RD and reversal learning errors; however, such a trend cannot survive multiple comparison correction.

To test whether these results generalized to other tasks where pre-potent responding and impulsive decision making lead to high error rates, we examined the relationship between performance on go/no-go and UF microstructure. Regression models were constructed to predict performance on the go/no-go task. After controlling for gender, neither UF nor ILF microstructure significantly predicted d′ on the go/no-go task (*p’s* > 0.20). Regression model data are presented in the Supplementary Table S2.

### Control Tract: the Inferior Longitudinal Fasciculus

Regression models were again constructed to predict performance on the reversal phase and performance on the acquisition phase of the IGT. The models were analogous to those constructed for the UF; predictors included gender (control variable) and bilateral ILF white matter indices (MD and AD). Regression model data are presented in **Table 3**. Based on the regression models, no relationships were found between ILF microstructure and performance on either phase of the IGT (*p’s* > 0.37). Similarly, there was no relationship found between go/no-go performance and any of the ILF indices (*p’s* > 0.90). Go/no-go regression data are presented in the Supplementary Table S2. In summary, no relationship was found linking microstructural properties of the ILF to performance on the IGT+reversal task or the go/no-go task.

## Discussion

The general function of association white matter pathways is to reciprocally connect gray matter loci in order to establish a temporally and spatially defined network that creates a unified, higher level function (Rolheiser et al., 2011). In this study, we examined an association white matter tract called the UF, which creates a monosynaptic pathway connecting portions of the medial and anterior temporal lobes to ventral portions of the frontal lobe (Ghashghaei and Barbas, 2002; Ghashghaei et al., 2007). Our review of the literature (Von Der Heide et al., 2013) implicated the UF in certain types of long-term memory retrieval (for a review, see Table 1 in Olson et al., under revision), as well as memory-based decision making (e.g., Bussey et al., 2002). During reversal learning, one must integrate long-term memories about prior good and bad choices with value computations in order to predict the optimal future choice. Thus, we predicted that individual variability in UF microstructure would predict reversal learning performance in healthy individuals. Our results provide support for this contention, as variability in the left UF predicted reversal learning errors. There appears to be some selectivity for this finding, because our evaluation of a control tract, the ILF, and a control task, go/no-go, provided no evidence of meaningful relationships with reversal learning and UF microstructure, respectively. The fact that our finding was left lateralized is consistent with existing findings on the UF, as findings are most commonly observed in the left hemisphere (for a review, see Table 1 in Olson et al., under revision).

Our findings were predicted by a study performed in macaques by Rudebeck et al. (2013), detailed in the Introduction. The authors speculated that reversal learning might rely on the integrated function of a hippocampal-amygdala-OFC circuit (Rudebeck et al., 2013). This finding is controversial because it stands in contrast to a robust literature showing reversal learning deficits after lesions to the OFC (Fellows and Farah, 2003; Frank and Claus, 2006; Fellows, 2011; Izquierdo and Jentsch, 2012). In humans, naturally occurring lesions will vary in size, location, and the degree to which white matter is damaged. It is probable that in many instances of human OFC damage, the entire hippocampal-amygdala-OFC circuit is compromised by concomitant white matter damage.

A similar argument has been made regarding the role of the amygdala in reversal learning. The same group (Rudebeck and Murray, 2008) conducted a related study in macaques, but focused on the amygdala. Prior studies had shown that aspiration lesions made to the amygdala led to reversal learning deficits (Schwartzbaum and Poulos, 1965; Aggleton and Passingham, 1981; Izquierdo and Murray, 2004). However, Rudebeck and Murray (2008) found that more refined excitotoxic lesions to the amygdala did not produce classic reversal learning deficits. Finally, a large literature, dating back decades, has reported that hippocampal lesions do not affect the acquisition of associations, but such lesions have a devastating effect on performance after reversal (reviewed in Shohamy et al., 2009). Similar deficits have been reported in rodents, monkeys, and humans following ablative lesions, as well as excitotoxic lesions. However, as is often the case, counter-evidence also exists suggesting that the hippocampus is also involved in the initial acquisition of associations (Henke, 2010).

Other neural regions also undoubtedly participate in reversal learning. For instance, the ventral striatum is thought to be involved in the computation of prediction error, the error signal that is generated to compute the difference between expected versus received rewards during learning (Li et al., 2011).

### Reversal Learning in Clinical Populations

Reversal learning deficits have been observed across a range of disorders. For instance, deficits at the reversal stage of learning tasks have been observed in several varieties of drug addiction, in both rodents and humans (Jentsch et al., 2002; Ersche et al., 2008; Schoenbaum and Shaham, 2008; de Ruiter et al., 2009; Stalnaker et al., 2009; Ortega et al., 2013). It has been speculated that drug addiction is associated with impairments in cognitive flexibility that make it difficult to inhibit pre-potent responses and update internal models with new stimulus–outcome mappings (Izquierdo and Jentsch, 2012).

Increased perseveration in reversal learning has also been observed in psychiatric disorders ranging from attentional disorders (e.g., ADHD) to disorders of emotion and social comportment, such as oppositional defiant disorder, conduct disorder, and psychopathy (Kempton et al., 1999; Itami and Uno, 2002; Mitchell et al., 2002; Budhani and Blair, 2005; Budhani et al., 2006; McKirdy et al., 2009; Roiser et al., 2009; Dickstein et al., 2010). The impulse control disorders are noteworthy because several studies have reported that individuals with conduct disorder and/or psychopathic traits have compromised amygdalae and OFCs (Blair, 2007), regions linked by the UF. Moreover, a small but consistent literature is emerging showing altered UF microstructure in individuals with psychopathic traits as compared to matched healthy controls (Craig et al., 2009; Passamonti et al., 2012; Sarkar et al., 2013). We speculate that difficulties in responding to changed reward and punishment contingencies underlie the relationship between psychopathic traits and altered UF microstructure. We further speculate that gross aberrations in this tract would lead to pathologically poor memory-based decision making, where memories can be retrieved and decisions made, but the two processes are poorly integrated.

### Limitations

It is important to bear in mind that white matter does not produce behavior; the electrical activity of neurons produces behavior. White matter provides the communication system that links groups of neurons to other groups of neurons. Thus, the information transmission properties of any given white matter tract are closely aligned with the function of the gray matter regions that it connects (Passingham et al., 2002).

Because the UF is a large white matter bundle linking functionally discrete regions of the temporal lobe (e.g., the uncus, entorhinal cortex, amygdala, perirhinal cortex, temporal pole, and possibly anterior hippocampus) to discrete regions of the frontal lobe (the lateral OFC and BA 10), it is plausible that the UF serves as the information conduit for a family of cognitive processes related to the functions of these regions. Our study was not designed to test the finer-grained question of whether white matter from particular cortical subregions underlies the observed association with reversal learning, but this will be important to examine in subsequent studies.

Another limitation is that our findings were only apparent in AD. AD measures the primary direction of diffusion within each voxel. It is thought to reflect properties such as axonal diameter, myelination (more specific), and axonal damage (less specific; RD has been shown to be more associated with axonal damage due to transection; Alexander et al., 2007; Feldman et al., 2010). We also observed an effect in MD; however, after correcting for multiple comparisons, this effect was no longer significant. Interpreting our findings from a detailed neurobiological standpoint is difficult. Our sampling resolution of approximately 2 mm × 2 mm × 2 mm is coarse; we are capturing far more than simply axons in our analysis. These different tissues and cell types give rise to certain architectural characteristics for each voxel, and it is clear that this architecture plays a role in the behaviors that we are observing. Beyond this, the variability that makes up the distribution of cells in each voxel, and the contribution of this to different DTI indices, is largely unknown.

Last, although we did observe variability in participants’ reversal learning performance, our cohort was more homogeneous than the U.S. population at large, since our participant pool consisted largely of Temple University undergraduate and graduate students. It is likely that our results would have been more robust if we had studied a population with greater variance in the measures of interest, such as individuals with conduct disorder, addiction, or ADHD.

## Conclusion

Research utilizing diffusion imaging techniques is still heavily focused on white matter alterations across clinical populations. Studies restricted to neurologically normal populations, and especially the investigation of individual differences among healthy individuals, are still rather rare. In this study, we demonstrate that individual differences in white matter microstructure are able to predict differences in reversal learning performance among a cohort of healthy young adults. We found that microstructural differences specific to the left UF predicted the number of errors committed on a reversal learning task. Therefore, the propensity to flexibly adapt to changing stimulus contingencies seems to be linked to microstructural properties of the UF. Portions of the hippocampus, amygdala, and OFC may form a circuit that allows associative learning to modulate our expectations about upcoming rewards and punishments, thus modulating decision making (Schoenbaum et al., 1998). The UF constitutes the “wires” of the circuit, allowing behavior to be modified by value-laden experiences. Future research should continue to investigate the specificity of this finding and should aim to expand our findings to clinical populations, particularly those characterized by deficiencies in cognitive flexibility.

## Conflict of Interest Statement

The authors declare that the research was conducted in the absence of any commercial or financial relationships that could be construed as a potential conflict of interest.
