# Supplementary material for: Fronto-temporal white matter connectivity predicts reversal learning errors
Source: Front Hum Neurosci. 2015 Jun 18;9:343. doi: 10.3389/fnhum.2015.00343 (PMC4471733; doi:10.3389/fnhum.2015.00343)
Supplement: Supplementary file 1 [file Data_Sheet_1.DOCX]

**Supplementary material.**

**Table S1.** Summary of multiple linear regression models predicting age.

|  |  | Uncinate Fasciculus | | | |  | Inferior Longitudinal Fasciculus | | | |
| --- | --- | --- | --- | --- | --- | --- | --- | --- | --- | --- |
| Dependent variable | Predictor variables | *β* | *t*-value | *F* | *R^2^* |  | *β* | *t*-value | *F* | *R^2^* |
| Age |  |  |  | 0.46 | 0.03 |  |  |  | 0.87 | 0.06 |
|  | Left AD | 0.20 | 0.92 |  |  |  | -0.12 | 0.67 |  |  |
|  | Right AD | -0.05 | 0.26 |  |  |  | -0.19 | 1.08 |  |  |
| Age |  |  |  | 0.12 | 0.01 |  |  |  | 1.01 | 0.06 |
|  | Left MD | 0.09 | 0.42 |  |  |  | -0.17 | 0.95 |  |  |
|  | Right MD | -0.09 | 0.41 |  |  |  | -0.17 | 0.93 |  |  |
| Age |  |  |  | 0.19 | 0.01 |  |  |  | 0.78 | 0.05 |
|  | Left RD | 0.40 | 0.20 |  |  |  | -0.18 | 0.96 |  |  |
|  | Right RD | -0.12 | 0.61 |  |  |  | -0.08 | 0.43 |  |  |
| Age |  |  |  | 0.38 | 0.03 |  |  |  | 0.15 | 0.01 |
|  | Left Streamlines | 0.09 | 0.30 |  |  |  | -0.05 | 0.20 |  |  |
|  | Right Streamlines | -0.21 | 0.75 |  |  |  | -0.06 | 0.21 |  |  |

AD: axial diffusivity, MD: mean diffusivity, RD: radial diffusivity, Streamlines: number of reconstructed tractography streamlines, *β*: standardized regression coefficient.

**Table S2.** Summary of multiple linear regression models predicting individual differences in performance on the go/no-go task.

|  |  | Uncinate Fasciculus | | | |  | Inferior Longitudinal Fasciculus | | | |
| --- | --- | --- | --- | --- | --- | --- | --- | --- | --- | --- |
| Dependent variable | Predictor variables | *β* | *t*-value | *F* | *R^2^* |  | *β* | *t*-value | *F* | *R^2^* |
| Go/no-go d’ |  |  |  | 1.74 | 0.21 |  |  |  | 0.35 | 0.05 |
|  | Gender | 0.47 | 1.98 |  |  |  | 0.24 | 0.94 |  |  |
|  | Left AD | 0.54 | 1.91 |  |  |  | -0.03 | 0.12 |  |  |
|  | Right AD | -0.39 | 1.57 |  |  |  | -0.003 | 0.01 |  |  |
| Go/no-go d’ |  |  |  | 0.44 | 0.06 |  |  |  | 0.40 | 0.06 |
|  | Gender | 0.20 | 0.79 |  |  |  | 0.27 | 1.07 |  |  |
|  | Left MD | -0.07 | 0.21 |  |  |  | -0.09 | 0.39 |  |  |
|  | Right MD | -0.06 | 0.22 |  |  |  | 0.02 | 0.09 |  |  |
| Go/no-go d’ |  |  |  | 0.73 | 0.10 |  |  |  | 0.70 | 0.10 |
|  | Gender | 0.11 | 0.47 |  |  |  | 0.23 | 0.97 |  |  |
|  | Left RD | -0.30 | 0.99 |  |  |  | -0.17 | 0.74 |  |  |
|  | Right RD | 0.11 | 0.37 |  |  |  | -0.10 | 0.42 |  |  |
| Go/no-go d’ |  |  |  | 0.80 | 0.11 |  |  |  | 1.08 | 0.14 |
|  | Gender | 0.21 | 0.96 |  |  |  | 0.22 | 1.01 |  |  |
|  | Left Streamlines | 0.25 | 0.69 |  |  |  | 0.41 | 1.44 |  |  |
|  | Right Streamlines | -0.01 | 0.04 |  |  |  | -0.28 | 0.98 |  |  |

AD: axial diffusivity, MD: mean diffusivity, RD: radial diffusivity, Streamlines: number of reconstructed streamlines, *β*: standardized regression coefficient.

**Table S3.** Summary of multiple linear regression models predicting individual differences in performance on the acquisition and reversal phases of the IGT.

|  |  | Uncinate Fasciculus | | | |  | Inferior Longitudinal Fasciculus | | | |
| --- | --- | --- | --- | --- | --- | --- | --- | --- | --- | --- |
| Dependent variable | Predictor variables | *β* | *t*-value | *F* | *R^2^* |  | *β* | *t*-value | *F* | *R^2^* |
| Acquisition Net Score |  |  |  | 0.47 | 0.05 |  |  |  | 0.13 | 0.01 |
|  | Gender | 0.10 | 0.54 |  |  |  | 0.09 | 0.45 |  |  |
|  | Left RD | 0.22 | 1.05 |  |  |  | -0.10 | 0.51 |  |  |
|  | Right RD | -0.18 | 0.87 |  |  |  | 0.04 | 0.20 |  |  |
| Acquisition Net Score |  |  |  | 0.51 | 0.05 |  |  |  | 0.68 | 0.07 |
|  | Gender | 0.04 | 0.24 |  |  |  | 0.11 | 0.60 |  |  |
|  | Left Streamlines | -0.21 | 0.74 |  |  |  | -0.30 | 0.11 |  |  |
|  | Right Streamlines | 0.33 | 1.16 |  |  |  | 0.27 | 1.03 |  |  |
| Reversal Errors |  |  |  | 1.41 | 0.13 |  |  |  | 1.43 | 0.13 |
|  | Gender | -0.25 | 1.41 |  |  |  | -0.16 | 0.88 |  |  |
|  | Left RD | -0.34 | 1.71 |  |  |  | -0.28 | 1.46 |  |  |
|  | Right RD | 0.21 | 1.10 |  |  |  | -0.07 | 0.37 |  |  |
| Reversal Errors |  |  |  | 0.60 | 0.06 |  |  |  | 0.43 | 0.04 |
|  | Gender | -0.20 | 1.10 |  |  |  | -0.18 | 0.95 |  |  |
|  | Left Streamlines | -0.10 | 0.35 |  |  |  | -0.12 | 0.46 |  |  |
|  | Right Streamlines | 0.22 | 0.78 |  |  |  | 0.13 | 0.50 |  |  |

IGT: Iowa Gambling Task, RD: radial diffusivity, Streamlines: number of reconstructed tractography streamlines, *β*: standardized regression coefficient.
